# Optimisation of pockels effect in poled amorphous waveguides for efficient electro-optic modulation

**DOI:** 10.1007/s00340-025-08477-0

**Published:** 2025-05-09

**Authors:** Sirawit Boonsit, Vasileios Mourgelas, Lara Karam, Milos Nedeljkovic, Nadege Courjal, Marc Dussauze, Ganapathy Senthil Murugan

**Affiliations:** 1 Optoelectronics Research Centre, University of Southampton, Southampton, SO17 1BJ UK; 2 Institut Des Sciences Moléculaires, Université de Bordeaux, Cedex 33405 Talence, France; 3 FEMTO-ST TEMIS, 15B Avenue Des Montboucons, Cedex 25030 Besancon, France

## Abstract

The induced second-order optical nonlinearity (SONL) in amorphous materials, such as silica glasses, has been extensively studied but remains significantly weaker compared to crystalline materials. Recent advancements demonstrated a remarkable induced $\chi_{xxx}^{\left( 2 \right)}$ value of 29 pm/V in amorphous sodo-niobate thin films (Na_2_O:Nb_2_O_5_) using a patterned thermal poling technique. In contrast to standard electro-optic single-crystalline materials, such as lithium niobate, thermally poled amorphous thin films exhibit a unique spatial distribution of $\chi^{\left( 2 \right)}$ nonlinearity, due to the structured electrodes poling process. This necessitates an advanced modelling approach tailored to poled amorphous materials. This study presents a theoretical analysis of the sodo-niobate dielectric permittivity tensor, then applies it to the design of electro-optic modulators using numerical simulations, to identify the optimal device geometry, device orientation, fabrication process, and poling configuration. Experimental parameters were included in the simulations to ensure design compatibility with fabrication. The optimized device configuration achieved a voltage-length product (V_π_L) of 3.87 V.cm. These designs establish poled sodo-niobate waveguides on SiO_2_ as a promising amorphous platform with a significant induced SONL response and practical fabrication potential for future electro-optic modulator applications.

## Introduction

Over the past decade, photonic integration technologies have advanced significantly to meet high demand for complex functionalities on a chip scale. A critical component in such systems is the external phase modulator. Traditionally, optical modulation has relied on bulky and complex benchtop setups [1], primarily suited for laboratory environments. However, the increasing need for portable and compact optical systems has driven the development of miniaturized electro-optic modulators (EOMs) for on-chip integration. These devices are essential in various applications, such as, telecommunications [2], optical sensing [3, 4] and quantum photonics [5]. EOMs manipulate optical signals by exploiting the electro-optic properties of specific materials under the influence of an electric field [6], inducing changes in the refractive index of these materials and thus altering the optical path length of the modulated signal. This trend towards miniaturization aligns with the broader paradigm shift towards lab-on-a-chip technologies [7].

A key challenge in miniaturizing EOMs lies in the selection and fabrication of suitable materials exhibiting the desired electro-optic properties. Crystalline materials such as lithium niobate (LiNbO_3_) [8] and barium titanate (BaTiO_3_) [9] are well-known for their strong electro-optic response. Nevertheless, despite their excellent nonlinear properties, crystalline materials encounter notable fabrication challenges stemming from their hardness, chemical inertness, and anisotropic nature [10]. In contrast, amorphous materials offer greater flexibility in waveguide fabrication due to their isotropic optical characteristics, such as consistent refractive indices and etching rate across all directions. This isotropy simplifies photolithographic patterning, plasma etching, and waveguide structuring, leading to highly uniform devices [11]. While lacking the inherent SONL, amorphous materials can be engineered to exhibit these properties through a technique called thermal poling. Recent advancements in inducing SONL in materials like sodium-doped niobium oxide (Na_2_O:Nb_2_O_5_) thin films have shown promising results (${\chi }^{\left(2\right)}=29 V.c{m}^{-1}$) [12, 13]. Moreover, Nb_2_O_5_ thin films, characterized by broad transparency, a high refractive index (~ 2.1–2.2 at 1550 nm), and significant induced ($\chi^{\left( 2 \right)}$), emerge as promising candidates for advanced waveguide platforms. The optimisation of EOM performance also involves the design and engineering of efficient modulator structures. Strategies such as optimizing slot waveguides [14, 15] with coplanar electrodes aim to enhance the interaction between the modulating electric field and the optical signal, thereby improving modulation efficiency and performance. While several studies have modelled the Pockels effect in LN-based EOMs, these designs primarily depend on the orientation of the LN crystal. The second-order nonlinear susceptibility tensor of LN is largely governed by on-diagonal components, allowing simplified refractive index distribution modelling [16]. In contrast, poled amorphous materials exhibit a different symmetry point group, characterized by off-diagonal elements in the permittivity tensor. These terms impact the EOM modulation, necessitating modelling to accurately predict device performance. In this study, we investigated the optimisation and comparison of Nb_2_O_5_ on silicon-based strip and rib waveguides with a nonlinearity induced through patterned poling to achieve the lowest possible V_π_L for electro-optic phase modulators. The dielectric permittivity tensor, based on the material point group symmetry, was incorporated into the simulation to estimate the refractive index distribution in the material. Both transverse electric (TE) and transverse magnetic (TM) mode polarisations were applied to each waveguide to assess the influence of poling direction on the optical field. Through detailed simulations, we analysed the interaction of waveguides subjected to patterned poling with TE and TM polarisation light at 1550 nm. Specifically, we explore the optimal conditions for TE mode modulation in strip waveguides of varying dimensions. The modulating electrode position was optimized to achieve a 1 dB/cm loss, and the modulating field was applied to induce changes in the mode effective index. The modulated phase and V_π_L of the waveguide were estimated to determine modulation efficiency. Additionally, we assessed the performance of waveguides and the impact of adding a silicon dioxide (SiO_2_) cladding layer. As a result, it is feasible to realize an EO modulator with a V_π_L of 2.59 V.cm (strip with top cladding) and 2.03 (rib with top cladding), which is about two orders lower than typically observed for induced SONL in amorphous waveguides [17]. Our modelling achieved promising modulation efficiency using poled amorphous material. This paves the way for practical fabrication methods that can exploit the versatility of amorphous poled materials in integrated photonic devices.

## Theory and background

We begin by reminding the reader of the second order optical properties of the poled amorphous sodo-niobate thin films reported by Karam et al. [12] that will be used to design our EO models. In these previous studies, a thermo electrical polarization imprinting process has been optimized on amorphous sodo-niobate films with thicknesses ranging from 0.5 to 2 µm. The poling-induced second order optical susceptibility of these centrosymmetric amorphous films was characterised by careful Maker Fringes Analysis and quantitative SHG microscopy. This has confirmed that a poling-induced second order optical response originated from an EFISH mechanism (Electric Field Induced Second Harmonic) attributed to charge gradient implantation within the amorphous niobate structure, leading to static electrical fields within the poled thin film.

Consequently, the second-order nonlinear susceptibility can then be written as,

1 $$\chi^{\left( 2 \right)} \left( { - 2\upomega ;\upomega ,\upomega } \right) = 3{\upchi }^{\left( 3 \right)} \left( { - 2\upomega ;\upomega ,\upomega ,0} \right) \cdot E_{int}$$

${E}_{int}$ is the internal static electric field induced during the poling treatment, ${\chi }^{\left(2\right)}$ and ${\chi }^{\left(3\right)}$ are second- and third-order nonlinear susceptibility, respectively.

The optimum geometry of the static electric field E_int_ was also determined by Karam et al.. Considering the plane (x,y) as the thin film surface, and that the z axis runs along the perpendicular direction, the effectiveness of the static field along the z axis ($\chi^{(2)}_{xxx}$) component was found to be 2 orders of magnitude lower than the planar components ($\chi^{(2)}_{xxx}$). This peculiarity originates from a Maxwell Wenger (MW) effect inducing charge accumulation at the film/substrate interface, which almost completely cancels out the effectiveness of the static electric field along the z axis. By contrast, the in-plane static electric field components are not affected by the MW effect, allowing efficient $\chi^{(2)}_{xxx}$ (or $\chi^{(2)}_{yyy}$) values which are independent of the film thickness to be obtained (within the range studied 0.5-2 µm).

Finally, using this finding as the input for our model, we can consider one unique static electrical field oriented along the x-axis direction. Such a uni-directional static field component allows the susceptibility of poled films to be considered from the point group $C_{\infty v}$. The second-order susceptibility tensor for such materials can be expressed as follows according to our model in ref. [13]:

2 $${\chi }^{\left(2\right)}=\left[\begin{array}{cccccc}{\upchi }_{xxx}^{\left(2\right)}& {\upchi }_{xyy}^{\left(2\right)}& {\upchi }_{xyy}^{\left(2\right)}& 0& 0& 0\\ 0& 0& 0& 0& 0& {\upchi }_{xyy}^{\left(2\right)}\\ 0& 0& 0& 0& {\upchi }_{xyy}^{\left(2\right)}& 0\end{array}\right]$$

While many studies on the second-order nonlinear (SONL) properties (${\chi }_{kji}^{\left(2\right)}$) of nonlinear materials utilize second harmonic generation (SHG) signals, practical applications, such as EOM, often report results in terms of the Pockels coefficient (${r}_{ijk}$). The EO coefficients were derived from the nonlinear optical susceptibility using the following relation [18]:

3 $${r}_{ijk}\left(-\omega ;\omega ,0\right)\approx -\frac{4\uppi {\chi }_{kji}^{\left(2\right)}\left(-2\omega ;\omega ,\omega \right)}{{\text{n}}_{i}^{2}{n}_{j}^{2}}$$

where ${\chi }_{kji}^{\left(2\right)}$ represents the second-order susceptibility obtained from second harmonic generation. Considering an isotropic material, the symmetric nature of the ${\chi }^{\left(3\right)}$ implies that $\chi_{xxx}^{\left( 2 \right)} = 3\chi_{xyy}^{\left( 2 \right)}$. Converting $\chi^{\left( 2 \right)}$ to the Pockels coefficient ($r$) can be achieved using Eq. (6), with the matrix tensor transposed to establish a relationship with the EO tensor form. Additionally, the index of coefficient ${r}_{ijk}$ can be contracted to ${r}_{i\alpha }$, with $\alpha$ replacing $jk$ where $\alpha = 1,2, \ldots ,6$.

4 $$r=\left[\begin{array}{ccc}{r}_{11}& 0& 0\\ {r}_{12}& 0& 0\\ {r}_{12}& 0& 0\\ 0& 0& 0\\ 0& 0& {r}_{12}\\ 0& {r}_{12}& 0\end{array}\right]$$

Thus, this tensor can be utilized to determine the refractive index change in the material in our EO calculations. The refractive index of the material can be explained using the index of ellipsoid, which describes the index profile (${\eta }_{ij}\left(0\right)+\Delta {\eta }_{ij}){x}_{i}{x}_{j}=1$). For an isotropic material, ${n}_{o}$ is the ordinary refractive index which remains uniform regardless of direction. As a result, the deformation of index of the ellipsoid can be expressed when the modulating voltage is applied along the x and z-axis, assuming ${E}_{y}=0$,

5 $$\left(\frac{1}{{n}_{o}^{2}}-{r}_{11}{E}_{x}\right){x}^{2}+\left(\frac{1}{{n}_{o}^{2}}-{r}_{12}{E}_{x}\right){y}^{2}+\left(\frac{1}{{n}_{o}^{2}}-{r}_{12}{E}_{x}\right){z}^{2}+2xz{(r}_{12}{E}_{z})=1$$

In amorphous material, where ${n}_{x}={n}_{y}={n}_{z}={n}_{o}$, refractive index change extends beyond the primary components to mixed terms, such as, x and z, suggesting that the major axes are not aligned with the x, y and z axes. Therefore, new axes must be defined considering both direction and magnitude. Assuming ${\upchi }_{{{\text{xxx}}}}^{{\left( {2} \right)}} {\text{ = 29 pm/V}}$, ${\upchi }_{{{\text{xyy}}}}^{{\left( {2} \right)}} { = 9}{\text{.67 pm/V}}$ [13], and *n*_o_ = 2.07 at wavelength of 1550 nm, we can estimate electro-optic tensor elements *r*_11_ and *r*_12_ using Eq. (5), giving values of 19.8 pm/V and 6.6 pm/V, respectively.

Traveling-wave electro-optic phase modulators leverage electro-optic materials where refractive indices vary in response to applied electrical fields. The change in real part of effective refractive index (${\Delta n}_{{{\text{eff}}}}$) induced by modulating the electric field allows for calculation of the phase shift (${\Delta }\phi$) using the following equation,

6 $$\Delta \phi =\frac{2\pi }{\lambda }\Delta {n}_{eff}L$$

Here, ${\uplambda }$ represents the operating wavelength, and *L* denotes the modulating length. The voltage required to induce a phase of ${\uppi }$ radians is referred to as the half-wave voltage (V_π_). When a Mach–Zehnder modulator employs phase shifters in both arms, it allows for independent modulation of the optical signal on each arm, typically in opposite directions, a configuration known as push–pull. If both phase shifters exhibit equal modulation efficiency and are driven symmetrically, a performance metric known as the Figure-of-merit (FOM) is defined in [19] as,

7 $${V}_{\pi }.L=\frac{\Delta V\lambda }{4\Delta {n}_{eff}}=\frac{\Delta V\lambda }{4(Real({n}_{eff}\left(V=0\right)-{n}_{eff}(V))}$$

where ${\Delta }V$ is the applied voltage to change the effective index of the material.

To optimize the Pockels effect in an Nb_2_O_5_ waveguide-based modulator, it is essential to ensure as much of the optical mode is in the Nb_2_O_5_ region (waveguide core) as possible while minimising optical losses. Simultaneously, it is crucial to maintain a compact optical mode to enable the realization of devices with significantly smaller footprints. In this work, we perform numerical simulations to optimise the geometry of an electro-optic phase modulator based on poled Nb_2_O_5_ strip and rib single-mode waveguides, aiming to achieve the lowest V_π_L possible.

## Modelling process, results and discussions

**Fig. 1:**
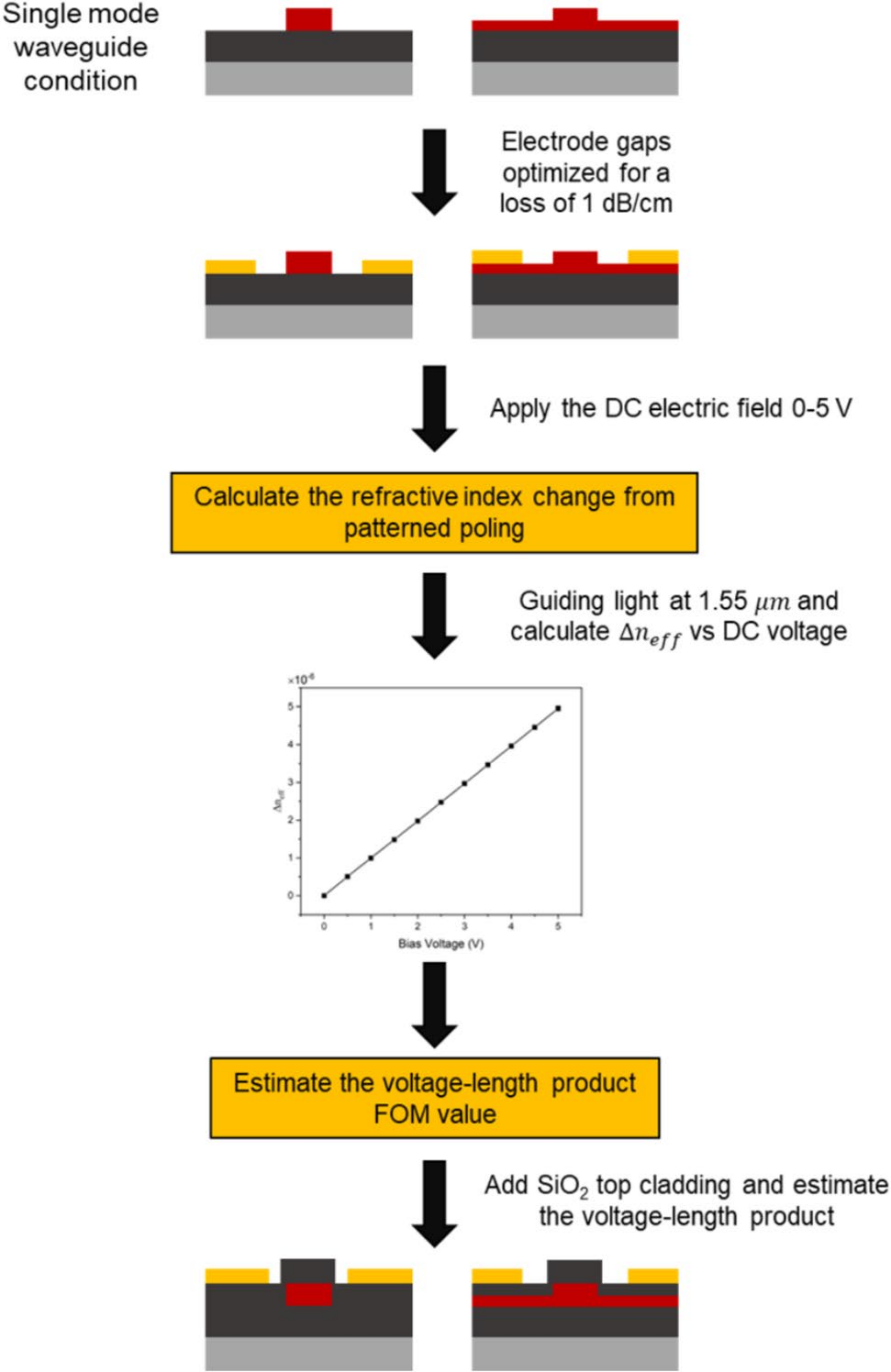
Schematic showing the simulation flow chart for optimising the modulation performance of induced EO material in two waveguide configurations (strip and rib)

In this section, we present a comprehensive simulation divided into six subsections, aimed at optimising two waveguide configurations for the design of a MZI based EOM using poled amorphous materials. The objective is to achieve the lowest possible V_π_L. Figure 1 provides an overview of the simulation schematic, starting with establishing the single-mode dimensions of strip waveguide (fully etched), and a rib waveguide (partially etched) for both fundamental TE and |TM modes. The next step involved optimising the electrode gap to minimise propagation loss of 1 dB/cm. Then, a voltage was applied across the electrode from anode to cathode (0–5 V). The perturbation of dielectric tensor was calculated according to Eq. 3. A waveguide propagation mode was then simulated at a wavelength of 1550 nm to obtain complex effective index (n_eff_). The shift in the real part of effective index was plotted against the applied voltage, allowing us to estimate the V_π_L product. Lastly, a SiO_2_ cladding layer was incorporated into the model to prevent direct contact between electrodes and calculate the voltage-length product. The details of each optimisation step and the corresponding results are discussed in the following subsections. The simulations are conducted using the finite difference eigenmode (FDE) module and the CHARGE module within the Ansys Optics Lumerical software package.

### Single-mode waveguide

**Fig. 2:**
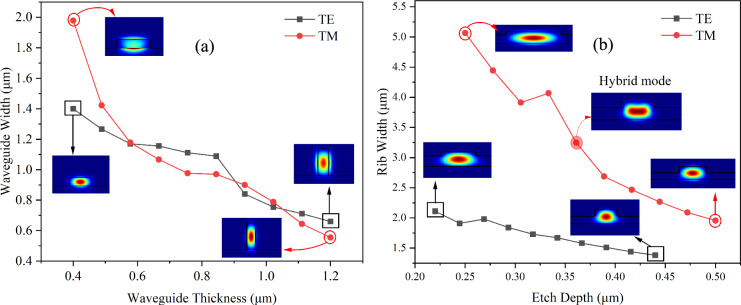
Single-mode dimension of **a** strip waveguides, and **b** rib waveguides, for TE and TM modes. The film thickness used for the rib waveguides is 0.88 µm and 1.0 µm for TE and TM (20−50% etch depth), respectively

The initial simulation targeted finding the dimensions of single-mode waveguides that support the fundamental TE and TM modes. The study involved varying the thickness of the strip within the range of 0.4–1.2 µm to find the maximum single mode width (i.e. the width at which the first higher order mode of the same polarisation appeared). This revealed that the single mode strip width for the TE mode varies between 0.6 and 1.4 µm, whereas for the TM mode it ranged from 0.5 to 2 µm, as shown in Fig. 2a. The plot also shows that at some thicknesses TE has a larger width, and at others the TM does.

**Fig. 3:**
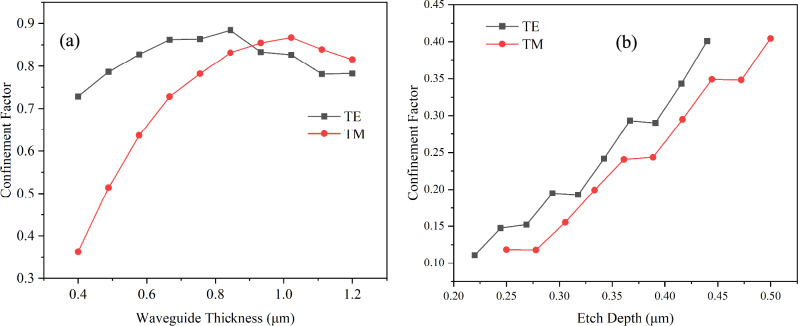
Confinement factor of **a** Strip waveguide **b** Rib waveguide for various single-mode waveguide conditions

Regarding the rib waveguide, the film thickness was selected by finding which single mode strip waveguide height achieved the highest field confinement value according to the data shown in Fig. 3a, resulting in a total film thickness of 0.88 µm for the TE mode and 1.0 µm for the TM mode. The etch ratios were varied from 25 to 50% of the total film thickness. The TE mode exhibited greater spatial extension in the x-direction, demanding narrower rib widths for single-mode guidance compared to the TM mode, which primarily extended along the z-axis. For instance, at an etch depth of 0.2 µm, the single-mode rib width for the TM_0_ mode was approximately 4 µm, while for the TE_0_ mode it was only 2 µm.

Notably, by using the single-mode searching algorithm, a polarisation factor is required to classify the modes as either TE or TM. Nevertheless, the red filled circle in Fig. 2b shows the hybrid mode (between TE and TM) that occurs when two orthogonally polarised modes with similar effective indices couple with each other [20]. This phenomenon can potentially affect the single-mode dimension of the waveguide. For instance, at the etch depth of 0.36 µm in the rib waveguide, the mode begins to convert as the rib width increases, leading to a change in the polarisation factor. Consequently, the single-mode width was observed to be slightly wider than expected (dip point). It is important to note that each data point in the following Figs. 3, 5, 8 corresponds to a specific combination of waveguide thickness and width for the strip waveguides, and a specific combination of thickness, width, and etch depth for rib waveguides, representing variations in the single-mode dimensions of the waveguide. This has been done to reduce the size of the search space, whilst conducting later device optimisation of the EO phase shifter.

### Confinement factor

In the following study, numerical simulations were performed to assess the modal confinement within the core region of single-mode waveguides. Confinement factor is a dimensionless parameter between 0 and 1 that quantifies the degree of optical power confined in the core relative to the surrounding cladding. Higher values signify stronger confinement of the guided mode.

For strip waveguides, it was found that the optimal dimensions for the TE mode fell within the range of approximately 0.8–0.9 µm waveguide thickness and width of 0.84 and 1.08 µm, resulting in nearly 0.90 confinement. The TM mode achieves a slightly lower 0.86, as shown in Fig. 3a. The TM mode exhibits more sensitivity to waveguide dimensions, for example, a thin waveguide has lower confinement.

In rib waveguides, the confinement factor for the TE mode within the core region (excluding the slab region) reached approximately 0.40 at the etch depth of 0.44 µm and the rib width of 1.38 µm (Fig. 3b). The confinement value for the TM mode was comparable. As etch depth and rib width increased, both modes had slightly higher confinement in the core region due to the thinner slab reducing the field extension into the slab region.

The confinement factor plots in the Fig. 3 are not perfectly smooth because each point corresponds to a unique combination of single-mode waveguide dimensions (thickness and width), rather than a single varying dimension.

### Optimising the electrode gap

**Fig. 4:**
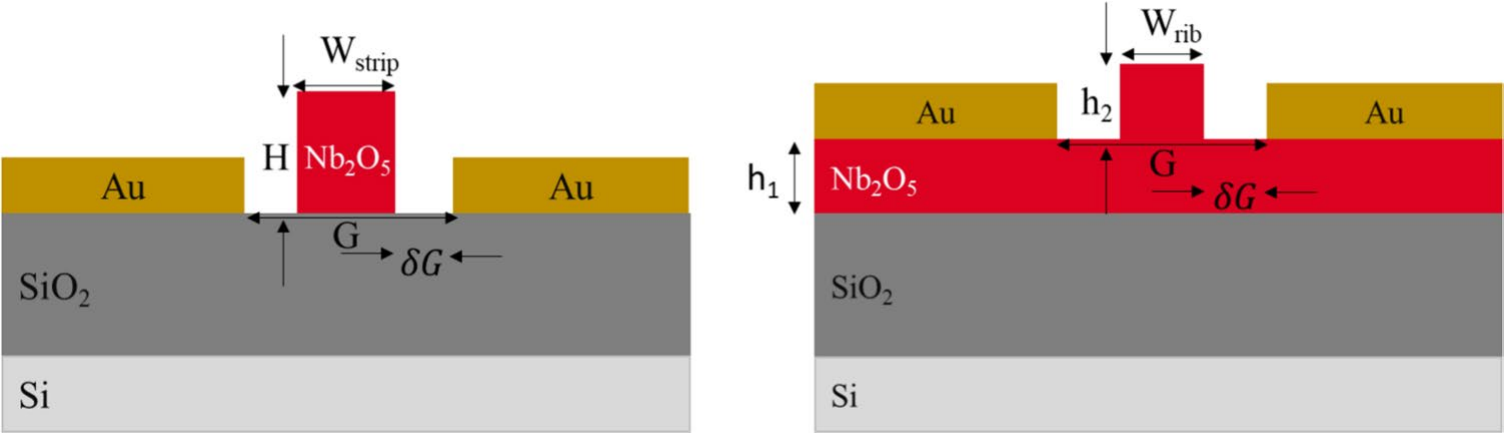
The diagram shows the model of a rib waveguide (left) and strip waveguide (right) with gold electrodes

**Fig. 5:**
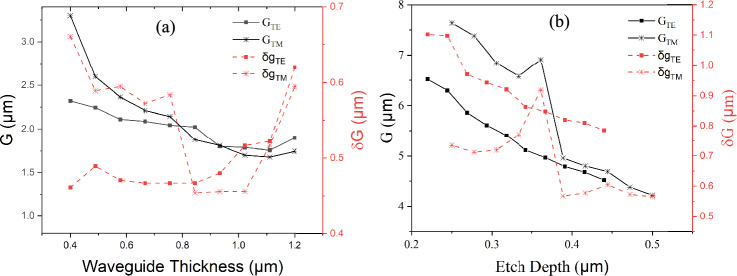
Plots showing the size of the gap between anode and cathode (G) that would give an excess waveguide loss of 1 dB/cm for **a** strip waveguides **b** rib waveguides for each single-mode waveguide dimension under investigation. In each figure the right-hand side y-axis shows the corresponding gap between the waveguide and electrode (δg)

**Fig. 6:**
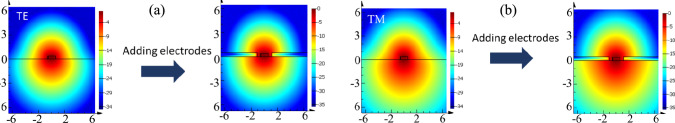
Optical electric field profiles for **a** TE mode **b** TM mode, in 0.4 μm thick strip waveguides, with and without electrodes. Electric field intensity is shown in log-scale, with the colorbar representing intensity strength corresponding to the expansion of the guided modes

Once the single-mode waveguide is tailored to confine either TE or TM modes effectively, the position of the gold electrodes relative to the waveguide core is chosen such that the metal absorption contributes a tolerable 1 dB/cm towards the waveguide propagation loss. Figure 4 illustrates the electrode geometry for the strip and rib configurations. Notably, the strip waveguide allows for a narrower gap between electrodes (G) (as well as a smaller gap between electrodes and waveguide (${\delta g}$) than the rib waveguide, which in turn leads to a more prominent electric field applied to the waveguide. The thicker slab in the rib waveguide led to weaker mode confinement, resulting in mode leakage, which can be absorbed by the metal, as illustrated in Fig. 5b. For the strip waveguide, the TM mode exhibited a broader spatial profile than the TE mode, particularly evident in a log-scale plot, leading to greater metal absorption for the TM mode. On the other hand, the data plotted for the rib waveguide clearly demonstrates that metal-induced loss affects the TE mode less compared to the TM mode (Fig. 6).

### Electric field overlap on the optical mode of a waveguide

**Fig. 7:**
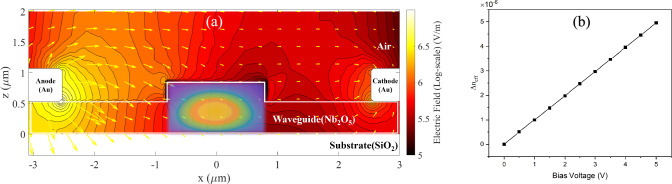
**a** Simulated optical field profile of the fundamental TE (2D surface plot) mode in the core region, alongside the modulating electric field distribution (contour plot) from anode to cathode in log-scale, with yellow arrows indicating field strength and direction. **b** Effective refractive index changes due to the applied voltage

In this study, the CHARGE module within Lumerical simulation was employed to investigate the electric field distribution across the waveguide, extending from the anode to the cathode (Fig. 7a). The applied voltage was varied from 0 to 5 V as shown in Fig. 7b. To determine the electric field, Poisson’s equation was solved, incorporating the low-frequency relative dielectric permittivity (${\in }_{r}$) of the materials. This parameter directly influences the magnitude of the electric field (E) within a material, ultimately impacting the modulation efficiency [21]. Here, a relative dielectric permittivity of 60 was used for the Nb_2_O_5_ material at a frequency of 100 Hz [22]. It is important to acknowledge that this value can vary depending on fabrication conditions.

The electric field profile obtained was subsequently incorporated into the mode calculation for each bias voltage. An example of the electric field profile with direction plotted as yellow arrows is shown in Fig. 7a. The perturbation of the inverse permittivity tensor due to applied electric field was implemented in the simulation based on Eq. (7) for patterned poling. The change of effective index of the fundamental TE mode in a rib waveguide is plotted with different voltages (0–5 V), as illustrated in Fig. 7b. For example, by calculating the ${\Delta n}_{{{\text{eff}}}}$ as shown in this graph, the voltage-length product can be estimated using the relation, $V_{\pi } \cdot L = \Delta V\lambda /4\Delta {\text{n}}_{{{\text{neff}}}} = \frac{{5 \times 1.55 \times 10^{ - 4} }}{{4 \times \left( {1.938027 - 1.937983} \right)}} = 4.39V \cdot {\text{cm}}$.

### Modulation efficiency

**Fig. 8:**
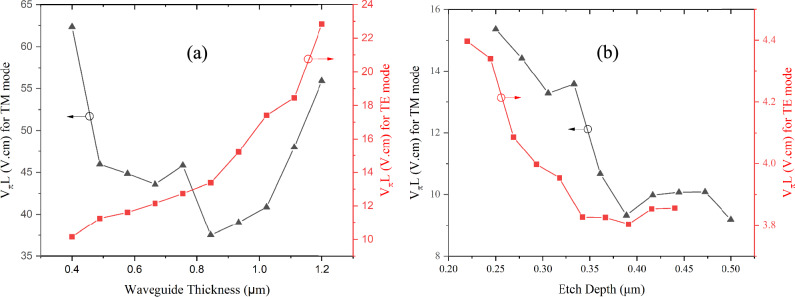
Voltage-length product of single-mode **a** Strip **b** Rib waveguides for TE and TM modes for patterned poling

The modulation efficiency of both strip and rib configurations is illustrated in Fig. 8. The findings indicate that the patterned poling offers significant efficiency for the TE mode, primarily due to the robust electro-optic interaction facilitated by the large induced electro-optic coefficient (r_11_). For strip waveguides, the V_π_L for the TE mode ranges between 10.1 and 22.8 V.cm (Fig. 8a). Conversely, the TM mode exhibits a weaker interaction between the modulating electric field (E_modz_) and the optical field of the TM mode (E_optz_), resulting in a lower modulation efficiency and a higher V_π_L (37.4-62.5 V.cm).

Furthermore, the TE mode in rib waveguides achieved lower V_π_L values (3.8–4.5 V.cm) compared to strip waveguides as shown in Fig. 8b. The significant permittivity contrast between Nb_2_O_5_ and its surrounding cladding at radio frequency would significantly reduce the coupling between the optical and electric field [23]. The presence of a slab region in the rib waveguide is crucial for facilitating an effective electric field within the rib. A thin slab can lead to a large voltage drop due to the higher permittivity contrast between the core region ($\in_{Nb2O5} = 60$) and the optical cladding ($\in_{SiO2} = 3.8$) [24]. The low-relative-permittivity of glass substrate can provide lower microwave index, enabling better velocity matching to the optical mode. This results in broader modulation bandwidth capabilities. This also eliminates the need for thick electrodes and buffer layers as in standard modulators [25]. However, a thicker slab can lower the optical confinement and result in wider electrode gaps. Once these parameters were optimized, the V_π_L of rib waveguides was lower than for strip waveguides. As expected, the V_π_L of the TM mode in rib waveguides is also higher (9.4–15.8 V.cm) due to the weaker EO interaction (r_12_) compared to TE mode (r_11_). Notably, the lowest V_π_L achieved in this work from a rib waveguide (3.86 V.cm) approaches the value of 1.29 V.cm reported for a thin-film lithium niobate (TFLN) Mach–Zehnder modulator at 1550 nm [16].

### *Impact of SiO*_*2*_* top cladding*

**Fig. 9:**
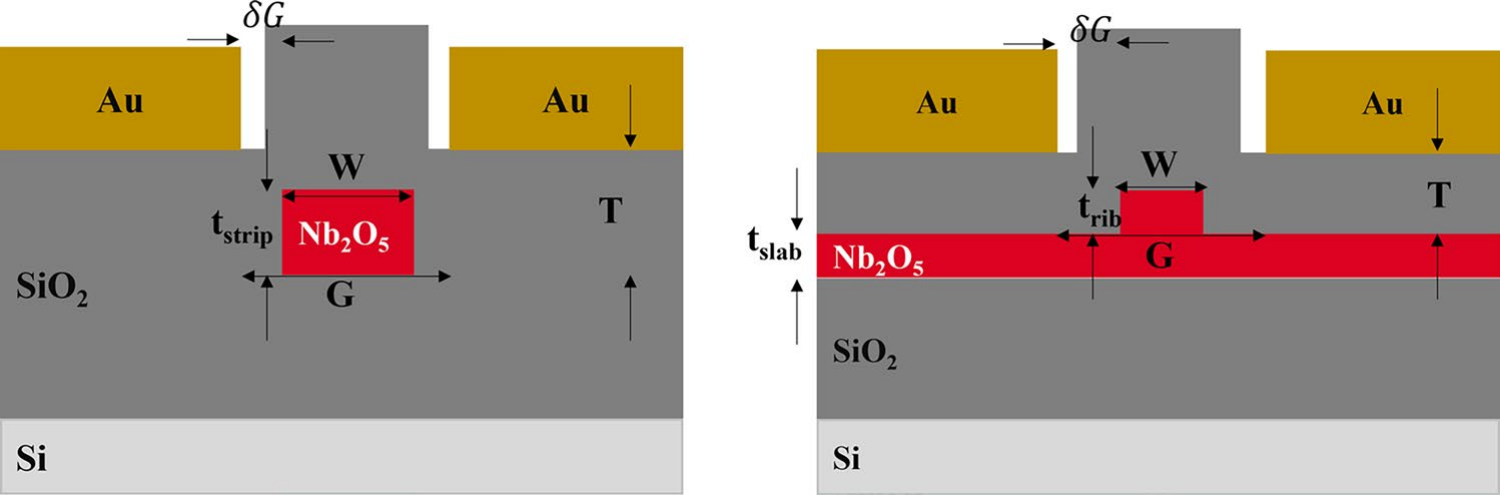
Diagram of Strip (left) and Rib (right) waveguide with SiO_2_ cladding of thickness T

**Fig. 10:**
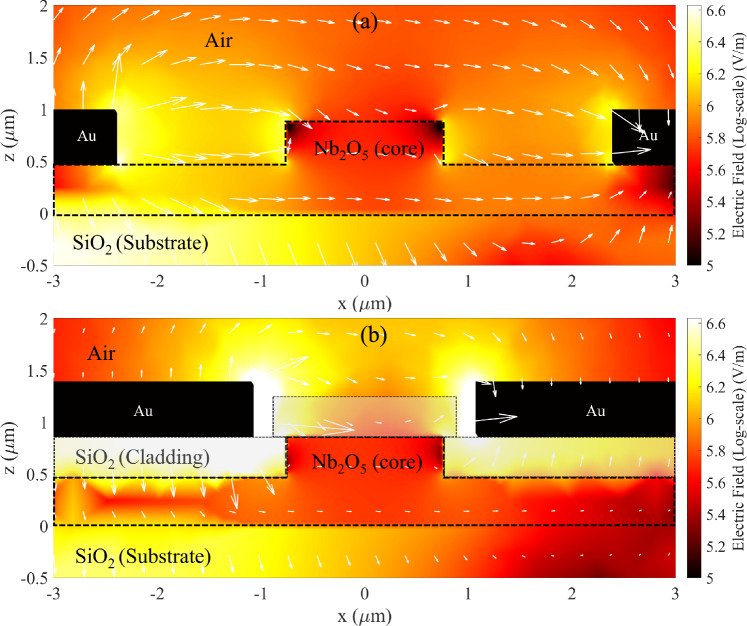
Log-scale plot of electric field distribution of rib waveguide **a** without and **b** with 400 nm SiO_2_ cladding, with white arrows indicating field strength and direction

The addition of a SiO_2_ top cladding serves multiple purposes in the design of the EOMs. Primarily, it serves to mitigate absorption losses resulting from metal electrodes (and contamination) and serves as a barrier to prevent direct contact of the metal from the top of the waveguide while reducing the effective gap and promoting the electric field interaction with the optical waveguide mode. Figure 9 shows the schematic of the waveguides and metal electrodes with the addition of a top cladding. In the case of the uncladded waveguides discussed in the above sections, a narrow electrode gap of approximately 2.32 μm yields a low loss less than 1 dB/cm for a strip waveguide. Conversely, in a rib waveguide to achieve a comparable level of low loss, a wider electrode gap of approximately 4.79 μm is required. The incorporation of 400, 600 and 900 nm SiO_2_ cladding layers enable the electrodes to be positioned closer to the rib waveguide, reducing the gap to 2.16, 1.84 and 1.82 μm, respectively, while maintaining the loss at 1 dB/cm. Despite this improvement, the EO response shows a slight reduction, with the V_π_L increasing to 4.15 V.cm with 400 nm cladding, compared to 3.87 V.cm in uncladded rib waveguide (7% decrease in modulation efficiency). This could be attributed to a weaker electric field strength in the rib region when the cladding is employed. Notably, as the SiO_2_ thickness increases, the V_π_L shows an increasing trend. This is primarily due to the electrode being positioned farther from the core along z-axis, which weakens the applied field strength despite the reduction in lateral gap. The resulting lower interaction between the optical and electric fields, as illustrated in Fig. 10.

**Fig. 11:**
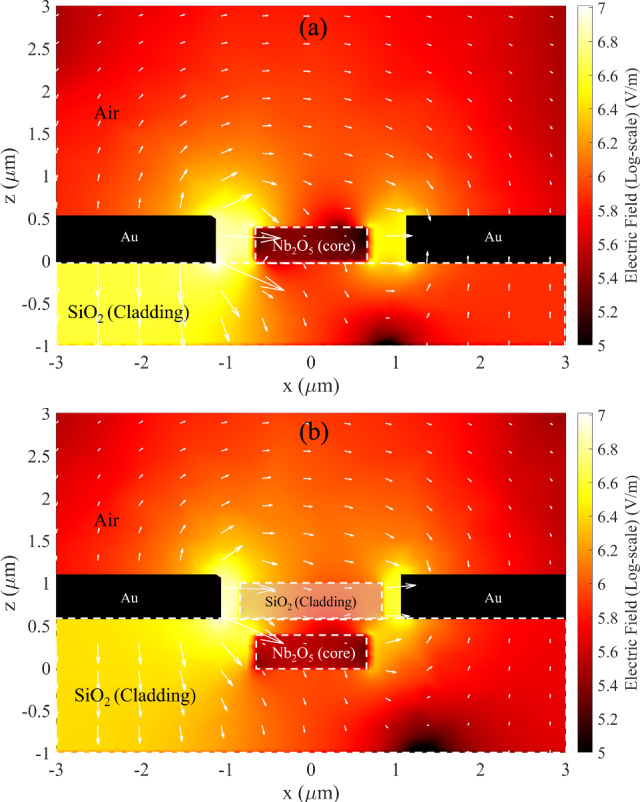
Log-scale plot of electric field distribution of Strip waveguide **a** without and **b** with 600 nm SiO_2_ cladding, with white arrows indicating field strength and direction

On the other hand, the addition of cladding layer has the opposite effect in strip waveguides compared to the rib waveguides. Considering two media with different dielectric constant, the electric displacement (D) must remain continuous at the interface. The electric field strength at the boundary can be described using this relation: $D={\varepsilon }_{1}{E}_{1}={\varepsilon }_{2}{E}_{2}$, where ε denotes dielectric constant of the material, and E represents the electric field strength within the material [26]. For the modulator without a SiO_2_ layer, the contrast of dielectric constant between air and Nb_2_O_5_ is significant. Since the electric field in Nb_2_O_5_ is inversely proportional to the dielectric constant, the field is predominantly concentrated in the air. This results in a limited electric field strength within the waveguides, as shown in Fig. 11a.

After adding a SiO_2_ layer, which has a higher dielectric constant (SiO_2_) compared to air [27], the electric field strength inside the core region increases. Consequently, the field interaction within the core region below the cladding layer is stronger. Figure 11b illustrates the electric field distribution inside the core, showing higher field strength compared to uncladded case. This enhanced interaction results in a reduction of the V_π_L from 10.10 to 8.19 V.cm, representing a 19% improvement in modulation efficiency for strip waveguides with 900 nm SiO_2_ cladding. The improvement in modulation efficiency with increasing cladding layer thickness is highlighting a key advantage of adding cladding layer for strip waveguides over rib waveguides. The effect of adding the SiO_2_ top cladding on the modulation efficiency is shown in Table 1.

**Table 1:** Summary of design parameters and voltage-length product of strip and rib waveguides with and without top SiO_2_ cladding for TE mode at optimised loss of 1 dB/cm. Key geometrical parameters and V_π_L are compared alongside representative crystalline EO modulators

Waveguide	t (µm)	W (µm)	T (µm)	Gap (µm)	δ_g_ (µm)	V_π_L (V.cm)
Strip (no top cladding)	t_strip_ = 0.40	1.40	–	2.32	0.46	10.10
Strip with top cladding			0.40	2.40	0.50	9.07
			0.60	2.22	0.41	8.35
			0.90	1.82	0.21	8.19
Rib (no top cladding)	t_rib_ = 0.39, t_slab_ = 0.48	1.51	–	4.79	1.64	3.87
Rib with top cladding			0.40	2.16	0.32	4.15
			0.60	1.84	0.16	4.51
			0.90	1.82^a^	0.15	6.06
Si/LN [28]	t_rib_ = 0.18, t_slab_ = 0.42	1.0	–	4.50	2.75	2.20
LNOI [29]	t_slab_ = 0.4, t_ridge_ = 0.2	1.3	1	7.0	2.85	2.37
TFLN [16]	t_rib_ = 0.18, t_slab_ = 0.22	1.1	0.50	5.0	1.95	1.29
BTO ridge [30]	t_ridge_ = 0.5	0.8	–	1.80	0.5	0.48

^a^The loss from this gap is negligible (0.07 dB/cm). However, the gap cannot be reduced further, as this would result in contact between the electrode and the top cladding due to δg being already low (0.15)

## Conclusion

We conducted numerical simulations of EOMs based on strip and rib waveguides. The primary objective was to optimise and compare various configurations (strip/rib, polarisation, electrode-waveguide gap) to achieve the lowest possible half-wave voltage-length product (V_π_·L), the figure-of-merit (FOM) of the modulator, while defining practical parameters for future fabrication and device characterisation. Unlike in LN, the $\chi^{\left( 2 \right)}$ distribution of poled amorphous films have a spatial distribution in point group C_∞*v*_ depending on the direction of the poling field. Here, the refractive index distribution of the poled sodo-niobate waveguide was derived based on the dielectric permittivity tensor, with the poling electric field aligned along x-, z-directions. This analysis revealed that the highest induced electro-optic response (r_11_) is highly sensitive to modulating electric field in x-direction, providing a guide to position the electrode to maximize modulation efficiency. Simulation results showed that a 0.39 µm thick and 1.51 µm wide rib waveguide exhibited the strongest modulation with a FOM of 3.87 V.cm. This was attributed to a pronounced interaction between the TE polarised light (at 1550 nm wavelength) and modulating electric field, corresponding to a high induced r_11_ of 19.8 pm/V observed in sodo-niobate thin film. By comparison, the best performing strip waveguide had more than twofold lower performance (10.1 V.cm). A cladding layer of SiO_2_ was added to improve the optical isolation while enabling crossover electrical interaction between various parts of the device. Varying SiO_2_ thickness to 400, 600 and 900 nm reduced absorption losses due to the metal electrodes and enabled narrower electrode gaps for a fixed waveguide loss of 1 dB/cm in both strip and rib waveguides. Strip waveguides exhibited a higher concentration of the electric field within the waveguide core and improved the modulation efficiency to 8.19 V.cm with a 900 nm cladding layer. In contrast, the addition of SiO_2_ cladding to rib waveguides reduced modulation efficiency slightly, achieving 4.15 V.cm with a 400 nm top cladding. These findings provide valuable insights for the design of poling configurations, such as patterned poling, depending on the modulating field component and polarisation used. For instance, patterned poling may be more suitable when employing coplanar electrodes due to its stronger interaction with the poling field. Overall, this study advances the design of EO phase modulators based on induced nonlinearity in amorphous Nb_2_O_5_ platform. With these optimised parameters, we are now well-positioned to proceed with photomask design for standard photolithography and dry etching, followed by a lift-off process for coplanar electrodes. This approach enables the fabrication of compact, thermally poled sodo-niobate waveguides for future modulation applications.

## Data Availability

The data for this work are accessible through the University of Southampton Institutional Research Repository "https://eprints.soton.ac.uk/ (accessed on 07 May 2025)".
